# 5hmC Immunohistochemistry: A Predictor of *TERT* Promoter Mutational Status in Follicular Thyroid Carcinoma?

**DOI:** 10.1369/00221554231190437

**Published:** 2023-07-24

**Authors:** Martin Hysek, Samuel L. Hellgren, Vincenzo Condello, Yiyi Xu, Catharina Larsson, Jan Zedenius, C. Christofer Juhlin

**Affiliations:** Department of Oncology-Pathology, Karolinska Institutet, Stockholm, Sweden; Department of Pathology and Cancer Diagnostics, Karolinska University Hospital, Stockholm, Sweden; Department of Oncology-Pathology, Karolinska Institutet, Stockholm, Sweden; Department of Pathology and Cancer Diagnostics, Karolinska University Hospital, Stockholm, Sweden; Department of Oncology-Pathology, Karolinska Institutet, Stockholm, Sweden; Department of Oncology-Pathology, Karolinska Institutet, Stockholm, Sweden; Department of Oncology-Pathology, Karolinska Institutet, Stockholm, Sweden; Department of Pathology and Cancer Diagnostics, Karolinska University Hospital, Stockholm, Sweden; Department of Molecular Medicine and Surgery, Karolinska Institutet, Stockholm, Sweden; Department of Breast, Endocrine Tumors, and Sarcoma, Karolinska University Hospital, Stockholm, Sweden; Department of Oncology-Pathology, Karolinska Institutet, Stockholm, Sweden; Department of Pathology and Cancer Diagnostics, Karolinska University Hospital, Stockholm, Sweden

**Keywords:** 5-hydroxymethylcytosine, follicular adenocarcinoma, immunohistochemistry, monoclonal antibodies, *TERT* promoter mutation, thyroid neoplasms

## Abstract

Telomerase reverse transcriptase (*TERT*) gene aberrancies correlate to adverse prognosis in follicular thyroid carcinoma (FTC). As loss of 5-hydroxymethylcytosine (5hmC) has been associated with *TERT* promoter mutations in papillary thyroid carcinoma, this study sought to analyze the levels of 5hmC in a cohort of follicular thyroid tumors with available *TERT* data. A total of 29 tumors (26 FTCs, 2 follicular thyroid tumors of uncertain malignant potential, and 1 oncocytic thyroid carcinoma) with known *TERT* promoter mutational status and *TERT* gene expression were assessed for 5hmC immunoreactivity using two antibodies (clones RM236 and 4D9.) Slides were analyzed using a semiquantitative scoring system. Of the 10 tumor cases with aberrant *TERT*, only 1 scored negative with both antibodies (1/10; 10%), whereas the remaining 9 cases (9/10; 90%) exhibited some positivity for at least one antibody. Of the 19 *TERT* wild-type tumors, no case was scored negative using RM236, and 2 cases (2/19; 11%) using 4D9. The differences between *TERT* promoter mutated and wild-type groups were non-significant. The sensitivity and specificity for 5hmC immunohistochemistry (IHC) to detect mutated cases were 10% and 100% (RM236) and 20% and 89% (4D9). Therefore, 5hmC IHC is not a sensitive marker for detecting *TERT* promoter mutations in follicular thyroid tumors.

## Introduction

Follicular thyroid tumors pose a diagnostic difficulty as the differentiation between follicular thyroid adenoma (FTA) and follicular thyroid carcinoma (FTC) is based on the identification of invasive properties using routine histology. To establish a correct diagnosis, the material often needs to be scrutinized thoroughly to identify areas of invasion. In the past years, activating hotspot mutations in the telomerase reverse transcriptase (*TERT*) gene promoter (denoted C228T and C250T) have been intimately coupled to FTC^1^ and subsets of follicular thyroid tumors of uncertain malignant potential (FT-UMPs), but are rarely observed in FTA. This genotype–phenotype correlation could therefore help in evaluating the true malignant potential of follicular thyroid tumors.^2^ Moreover, *TERT* promoter mutations and *TERT* gene expression are prognostic factors in thyroid cancer, as these events are closely associated with adverse histopathological factors and poor patient outcomes.^3–7^ As of this, the 2022 WHO classification of endocrine and neuroendocrine tumors acknowledges the role of *TERT* promoter mutations in highlighting cases at risk of disease dissemination,^8^ and more focus is currently diverted to developing robust screening tools to detect *TERT*-expressing tumors in clinical routine. Specifically, whereas *TERT* immunohistochemistry (IHC) is not yet considered a reliable method, studies using *TERT* mRNA in situ hybridization (ISH) have shown promising results.^9,10^

5-Hydroxymethylcytosine (5hmC) immunoreactivity is decreased in various malignant tumors, such as melanoma^11^ and glioma^12^ as well as in papillary thyroid carcinoma (PTC).^13,14^ 5hmC is produced when 5-methylcytosine is demethylated via ten-eleven translocation (TET) enzymes, and 5hmC may thus serve as an epigenetic marker of global de-methylation events across the genome. Oishi et al.^14^ recently showed an association between the loss of 5hmC and *TERT* promoter mutations in PTC, but to our knowledge, no efforts to predict *TERT* promoter mutational status by 5hmC IHC in FTCs have been published. The ability to predict *TERT* promoter mutational status based on an immunohistochemical marker would be an attractive solution for high-volume centers in which molecular work-up is not performed routinely, as this genetic aberrancy is so tightly coupled to thyroid carcinoma and poor-prognosis cases rather than benign tumors.

Therefore, this study aimed to evaluate whether loss of immunohistochemical 5hmC expression can predict *TERT* promoter mutations in a cohort of FTCs with established *TERT* promoter genotypes and *TERT* mRNA expression.

## Materials and Methods

### Cohort

A cohort of differentiated follicular cell-derived and oncocytic thyroid tumors which has previously been evaluated for *TERT* promoter mutations and *TERT* expression using qRT-PCR (Quantitative reverse transcription PCR) and ISH^10^ was chosen for 5hmC IHC analyses. The cohort consisted of 26 FTCs, 2 FT-UMPs, and 1 oncocytic thyroid carcinoma (OTC), of which 10 (7 FTCs, 2 FT-UMPs, and 1 OTC) showed *TERT* promoter mutations and *TERT* gene expression, whereas the remaining cases were *TERT* promoter wild-type and displayed absent *TERT* mRNA levels. A de-identified case with thyroid follicular nodular disease (TFND) was included as a non-tumorous positive control for both antibodies. A de-identified sample of normal testis tissue was used as a positive control of 5hmC immunoreactivity and to identify the best dilution of the 4D9 antibody. To test the validity of the antibody used in clinical routine, three de-identified malignant thyroid tumors with concurrent *BRAF* and *TERT* promoter mutations were included as negative controls for the antibody.

### 5mhC IHC (Clone RM236)

Formalin-fixed paraffin-embedded (FFPE) tissues from all cases were sectioned in 4-micrometer thick sections. The chosen sections all contained tumor tissue as well as adjacent normal thyroid tissue. After de-paraffinization in xylene and rehydration in alcohol, sections were subjected to heat-induced antigen retrieval using the Ventana Ultra CC1, pH 8.5 for 32 min. The staining was automated using the Ventana Ultra Benchmark methodology and a primary antibody (5hmC clone RM236, Abcam, Cambridge, UK) was applied at a dilution of 1:1000 for 32 min as used in our clinical routine practice. Visualization was performed using OptiView and slides were then counterstained using Mayer’s hematoxylin for 3 min.

The antibody was previously validated for use in IHC and in thyroid carcinoma.^15–17^

### 5hmC IHC (Clone 4D9)

Following de-paraffinization and rehydration of FFPE sections, heat-induced antigen retrieval was performed using a decloaking chamber (Biocare Medical, Pleasant Hill, CA) set for 5 min at 110C in citrate buffer pH 6 (article C-9999, Sigma-Aldrich, St. Louis, MO). For quenching of endogenous peroxidase, a 30-min incubation in 0.15% hydrogen peroxidase was performed at room temperature, followed by a 30-min blocking step using 1% bovine serum albumin (article A-4503, Sigma-Aldrich, MA). The primary 5hmC monoclonal antibody (HMC/4D9, Epigentek, NY) was diluted 1:2000 in Renoir Red diluent (article No. PD9004M, Biocare Medical, CA), before incubation at 4C overnight in a humidified chamber. The optimal dilution of the antibody was established through serial staining of normal testicular tissue in which we selected the dilution in which the nuclear 5hmC staining was clear-cut and diffuse without interfering background or cytoplasmic expression (data not shown). For detection, the Mach-1 Universal HRP-Polymer Kit was used (article MIU539L10, Biocare Medical, CA) according to the protocol provided by the manufacturer. The sections were counterstained in Mayer’s hematoxylin for 1 min, followed by dehydration with graded alcohols, xylene treatment, and coverslipped with Pertex (Histolab, Gothenburg, Sweden).

### 5hmC Dot Blot (Clone 4D9)

To further validate the 5hmC affinity of the antibody clone 4D9, we also performed dot blot analysis on two PTC cell lines, one with a known *TERT* promoter mutation (MDA-T32) and one with wild-type *TERT* promoter (MDA-T41), both purchased from the American Type Culture Collection, Manassas, VA. The cells were cultured in RMPI-1640 medium supplemented with 10% FBS and 1% non-essential amino acid at 37C and 5% CO_2_. The *TERT* promoter mutational status in both cell lines was verified by Sanger sequencing, and short tandem repeats genotyping was performed and matched to a previous publication.^18^ Pierce RIPA Buffer (Thermo Fisher Scientific, Waltham, MA) with 10% Protease Inhibitor Cocktail (Sigma-Aldrich, MO) and 1% Phenylmethanesulfonyl fluoride (Sigma-Aldrich, MO) was used for the preparation of PTC cell lysates.

**Figure A1.**
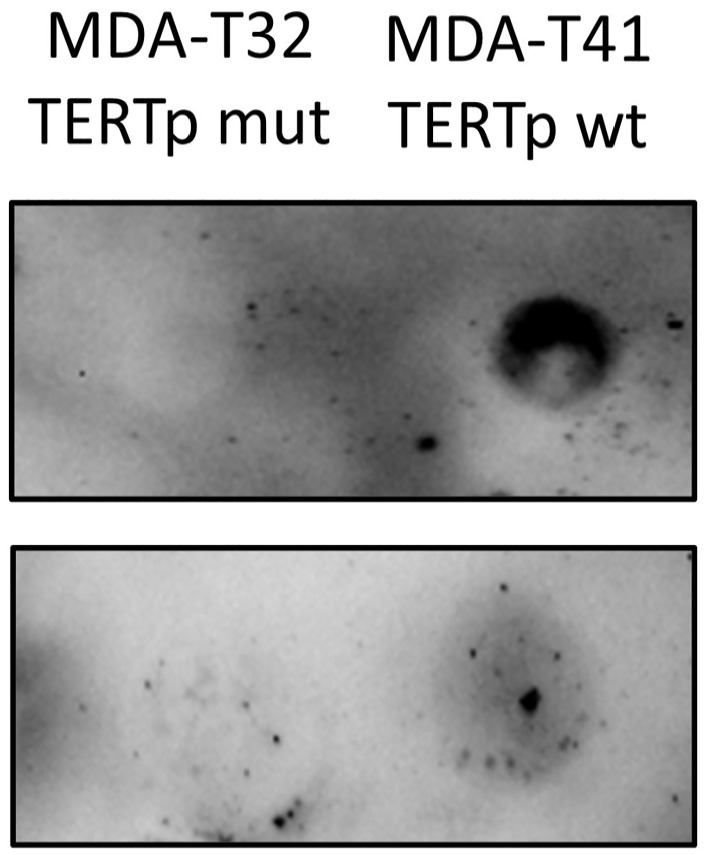
Dot blot analysis in papillary thyroid cancer (PTC) cell lines: PTC cell line protein lysates were blotted onto a membrane and exposed to the 5hmC antibody clone 4D9. Left: *TERT* promoter mutated PTC cell line MDA-T32 showing no reaction with the 5hmC antibody; right: *TERT* promoter wild-type PTC cell line MDA-T41 shows retained 5hmC expression. Top and bottom rows represent two independent analyses using different lysates. Abbreviation: *TERT*, telomerase reverse transcriptase.

After quantifications with BCA Protein Assay (Bio-Rad, Hercules, CA), 30 µg of lysates were spotted on a 0.2-µM nitrocellulose membrane following an indirect immunoassay procedure. After blocking in 5% milk diluted in tris-buffered saline-T (150 mM NaCl, 10 mM Tris-HCL, pH 7.5, 0.05% v/v Tween 20) for 20 min, the membrane was incubated with the primary antibody (1:1000 dilution), then with peroxidase-conjugated goat anti-mouse IgG (1:2000 dilution, Invitrogen, 62-6520). SuperSignal West Femto Maximum Sensitivity Substrate (Thermo Fisher Scientific, MA) was used for the detection of specific signals. A specific signal was observed in the *TERT* promoter wild-type cell line (MDA-T41) and no signal in the *TERT* promoter mutated cell line (MDA-T32; Appendix Fig. A1).

### Scoring Methodology

The stained slides were evaluated by two pathologists independently following the same semiquantitative scoring system as Oishi et al. for their study on PTC:^14^ 0 = complete lack of nuclear staining in tumor cells (i.e. negative); 1 = positive staining only in scarce tumor cells (1–9%); 2 = positive staining in 10–24% of tumor cells; 3 = positive staining in 25–74% of tumor cells; and 4 = diffuse and strong nuclear staining in ≥75% of tumor cells.

**Figure 1.**
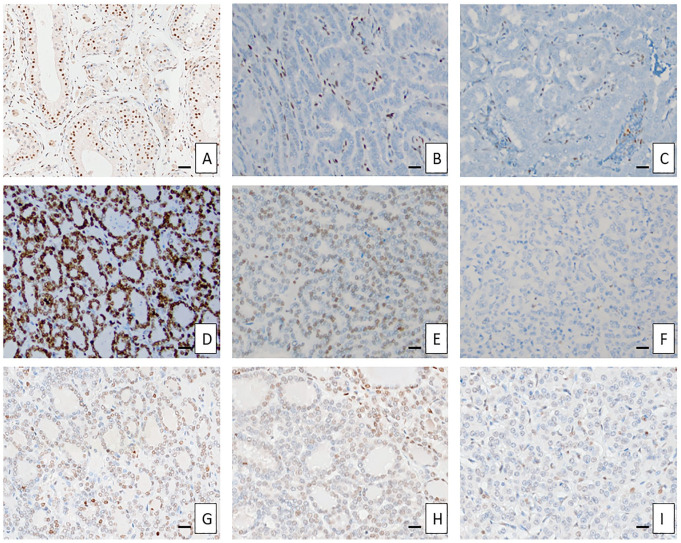
Different 5hmC staining patterns. Control tissues (Panels A to C): normal testicular tissue (100× magnification), manually stained with clone 4D6 (Panel A); negative stain in a *BRAF* + *TERT* promoter mutated oncocytic papillary thyroid carcinoma (PTC), Ventana clone RM236 (200×, Panel B); negative stain in a *BRAF* + *TERT* promoter mutated tall cell PTC, Ventana clone RM236 (200×, Panel C); Clone RM236 (D to F): Score 4 (Sample No. 28, a *TERT* promoter wild-type follicular thyroid carcinoma [FTC] without *TERT* mRNA expression, 200×, Panel D); Score 3 (Sample No. 4, a *TERT* promoter mutated FTC with established *TERT* mRNA expression, 200×, Panel E); Score 0 (Sample No. 8, a *TERT* promoter mutated follicular thyroid tumor of uncertain malignant potential with established *TERT* mRNA expression, 200×, Panel F). Clone 4D6 (G to I): Score 4 (Sample No. 28, 200×, Panel G); Score 3 (Sample No. 4, 200×, Panel H); Score 0 (Sample No. 8, 200×, Panel I). Bar A = 20 micrometers; bars B to I = 10 micrometers. Abbreviations: mRNA, messenger ribonucleic acid; *TERT*, telomerase reverse transcriptase.

Examples of different staining patterns are shown in Fig. 1.

### Statistical Analyses

Fisher’s exact tests were used to compare 5hmC expression and *TERT* promoter mutational status. Chi-square and Mann–Whitney *U* tests were used to compare clinicopathological features and *TERT* promoter mutational status. *p* values less than 0.05 were considered significant. Statistical computations were performed using RStudio v12.0 (Posit Software PBC, Boston, MA).

## Results

### Clinicopathological Features of the Study Cohort

A total of 29 tumors from 29 patients were included in this study. The mean age at diagnosis was 53.2 years and 72% of the patients were female. The tumors were between 15 and 100 mm in size (mean 44.8 mm) and we calculated an average proliferation index (Ki-67) of 5.9%. In five cases, distant metastases were identified. The comparisons between *TERT* promoter mutated and wild-type cases are summarized in Table 1.

**Table 1.** Clinicopathological Features of the Follicular and Oncocytic Thyroid Tumors Included in the Study Cohort.

Clinicopathological Parameters	*TERT* Promoter Mutated	*TERT* Promoter Wild-Type	Total	*p* Value
Number of cases	10	19	29	
Gender: female (*n*, %)	6 (60)	15 (79)	21 (72)	0.51
Age at diagnosis, years	65.4	46.7	53.2	0.49
Size, mm	47.2	43.6	44.8	0.73
Ki-67, %	7.3	5.1	5.86	0.06
Distant metastasis (*n*, %)	4 (40)	1 (5)	5 (17)	0.06

Abbreviation: *TERT*, telomerase reverse transcriptase.

### Evaluation of 5hmC Immunoreactivity Across the Tumor Cohort

For both antibodies, positive and negative controls were evaluated. The IHC for 5hmC yielded various results regarding staining intensity in tumor tissues, requiring us to implement the semiquantitative scoring system described in the Materials and Methods section based on nuclear staining in an estimated percentage of tumor cells. The staining outcomes and *TERT* gene status are detailed together with the histopathological diagnosis for each case in Table 2. The control case exhibiting TFND and the normal testicular tissue sample used as positive controls displayed a score of 4 for both antibody clones, as did all cases in the adjacent normal thyroid tissue whenever present on the same slide as the tumor tissue (data not shown). The three de-identified malignant thyroid tumors with concurrent *BRAF* and *TERT* promoter mutations (used as negative controls) all exhibited complete nuclear absence of 5hmC staining (Tier 0).

**Table 2.** Summarized Genetic and Expressional Cohort Data.

Sample No.	Diagnosis	*TERT* Mutation^a^	*TERT* Expression^a^	5hmC IHC (RM236) Score	5hmC IHC (4D9) Score
1	FTC	**C228T**	**0.65**	4	1
2	FTC	**C228T**	**1.00**	3	3
3	FTC	**C228T**	**5.28**	3	3
4	FTC	**C228T**	**14.93**	3	3
5	FTC	**C228T**	**1.01**	3	2
6	FTC	**C228T**	**1.23**	3	0
7	FTC	**C228T**	**2.57**	3	2
8	FT-UMP	**C228T**	**2.69**	**0** ^b^	**0** ^b^
9	FT-UMP	**C228T**	**10.86**	3	3
10	OTC	**C250T**	**5.37**	4	3
11	FTC	WT	0	4	4
12	FTC	WT	0	3	2
13	FTC	WT	0	3	2
14	FTC	WT	0	3	2
15	FTC	WT	0	4	4
16	FTC	WT	0	4	3
17	FTC	WT	0	3	3
18	FTC	WT	0	3	3
19	FTC	WT	0	3	1
20	FTC	WT	0	2	2
21	FTC	WT	0	4	3
22	FTC	WT	0	3	2
23	FTC	WT	0	4	4
24	FTC	WT	0	4	3
25	FTC	WT	0	4	4
26	FTC	WT	0	4	4
27	FTC	WT	0	4	0
28	FTC	WT	0	4	0
29	FTC	WT	0	4	1
30	TFND	Not known	Not known	4	4

*TERT* promoter mutational status (C228T = C>T point mutation at position 228; C250T C>T point mutation at position 250, WT = wild-type). Normalized *TERT* expression value (qRT-PCR), 5hmC IHC semiquantitative (0: <1%; 1: 1–9%; 2: 10–24%; 3: 25–74%; 4: >74%). Abbreviations: *TERT*, telomerase reverse transcriptase; IHC, immunohistochemistry; FTC, follicular thyroid carcinoma; FT-UMP, follicular thyroid tumor of uncertain malignant potential; OTC, oncocytic thyroid carcinoma; TFND, thyroid follicular nodular disease; qRT-PCR, quantitative reverse transcription PCR.

a Cases in bold had established TERT promoter mutations

b Case with loss of 5hmC expression.

### Comparing 5hmC IHC to TERT Promoter Mutational Status and TERT Expression

For the 4D9 clone, of the 10 tumors with an established *TERT* promoter mutation and *TERT* gene expression, 2 (20%) stained negative (Tier 0) whereas 8 cases exhibited positive staining in more than 1% of tumor cells (Tier 1 or above). Of the 19 *TERT* promoter wild-type cases, 2 (10.5%) demonstrated a negative 5hmC immunoreactivity, whereas 17 cases (89.5%) were seen with more than 1% positive tumor cells (Fisher’s exact test *p*=0.59). The sensitivity and specificity for global loss of 5hmC (clone 4D9) to detect *TERT* mutated cases were 20% and 89%, respectively (Table 3). For the RM236 clone, one of the *TERT* promoter-mutated tumors stained negative (Tier 0), whereas nine cases demonstrated a Tier 1–4 stain, compared with *TERT* promoter wild-type cases in which the corresponding numbers were 0 and 19, respectively (Fisher’s exact test *p*=0.35). The sensitivity and specificity for the RM236 clone to detect *TERT* promoter mutations were 10% and 100%, respectively (Table 3).

**Table 3.** Sensitivity, Specificity, and PPV/NPV of the Semiquantitative Scoring.

	5hmC IHC (4D9 Clone) Semiquantitative Score = 0	5hmC IHC (4D9 Clone) Semiquantitative Score = 1–4	5hmC IHC (RM236 Clone) Semiquantitative Score = 0	5hmC IHC (RM236 Clone) Semiquantitative Score = 1–4
Tumors with *TERT* mutation/expression	2	8	1	9
Tumors without *TERT* mutation/expression	2	17	0	19
Sensitivity, %	20		10	
Specificity, %	89		100	
PPV, %	50		100	
NPV, %	68		68	
Fisher’s exact test	*p*=0.59		*p*=0.35	

Abbreviations: IHC, immunohistochemistry; NPV, negative predictive value; PPV, positive predictive value; *TERT*, telomerase reverse transcriptase.

## Discussion

*TERT* promoter mutations and *TERT* expression are harbingers of worse clinical outcomes in patients with FTC, constituting important prognostic markers. Because it is certainly feasible to interrogate both DNA and RNA acquired from FFPE material in the clinical setting, the advent of an IHC marker with the ability to triage cases for further testing would of course be a valuable tool in the clinical setting, given the low cost and general availability across pathology laboratories. In this cohort of follicular thyroid tumors, we could not verify earlier observations made in PTC suggesting that a loss of 5hmC would reliably signify an underlying *TERT* promoter mutation. If accepting global loss (Score 0) as a pathogenic staining pattern, the sensitivity of the method reached 20% and 10% for the 4D9 and RM236 clones, respectively, in our series, whereas the specificity was rather low. Given the rather low percentages of follicular thyroid tumors exhibiting *TERT* promoter mutations in unselected series (around 10%), the method would require high specificity to minimize the risk of false positive cases.

In this cohort of follicular thyroid tumors and OTCs, we could not fully reproduce the observations of global loss of 5hmC immunoreactivity observed in PTCs with *TERT* promoter mutations.^13,14^ The discrepancies may in part be due to the different thyroid tumor types studied. Indeed, the inheritably different biology of a *BRAF* mutation compared with predominantly *RAS*-driven FTCs may affect the global epigenetic landscape differently in *TERT* promoter-mutated cases. Seok and Fan recently showed a relationship between *BRAF*-mutated PTC and reduced 5hmC levels, but no such correlation in other groups of follicular patterned thyroid tumors.^19^ The pathway linking *BRAF* mutations with lower levels of TET proteins and 5hmC is well established,^20^ whereas to our knowledge no such linkage has been examined for the more heterogeneous group of non-*BRAF*-driven thyroid neoplasms.

In the original publication in which PTCs were assayed, Oishi et al. used a single, polyclonal antibody, whereas we incorporated two different monoclonal anti-5hmC antibodies. However, our staining of *BRAF-*driven thyroid tumors with *TERT* promoter mutations gave rise to comparable results as Oishi and colleagues observed: a global loss of 5hmC (Fig. 1). Therefore, the discrepancies in the staining outcomes in follicular thyroid tumors in association with underlying genotypes are most likely due to intrinsic differences in tumor biology rather than methodological diversities. Furthermore, we saw no apparent differences in terms of sensitivity and specificity regarding the two antibody clones used in this study. Moreover, because we employed two different staining techniques (automated vs manual), this parameter is not believed to affect the outcome either.

*TERT* promoter mutations and *TERT* gene expression are not unequivocally needed for the global loss of 5hmC in thyroid cancer. There is much to be revealed regarding the biology of *TERT* promoter mutations in follicular thyroid tumors, as *TERT* protein expression itself may not be correlated to *TERT* promoter mutations and *TERT* mRNA expression^9^ as well as the recent observation of nuclear-specific *TERT* mRNA expression.^10^

*TERT* aberrancies may exhibit spatial heterogeneity in follicular thyroid tumors, which has been shown for *TERT* promoter mutations^21,22^ as well as for *TERT* mRNA expression visualized by ISH.^10^ It was therefore expected that some heterogeneity could be observed regarding the expression of 5hmC; it is however interesting that as many as half of all cases showed heterogeneous expression patterns—a finding that lacks a credible explanation.

Evaluating immunohistochemical staining where the required result is a negative staining pattern may not be without problems on its own. It is not always evident whether negative staining is due to technical mishaps or relates to a true biological reason. However, in the material examined in this study, the surrounding normal thyroid as well as stromal elements within the tumor tissue itself were available for internal control purposes, suggesting preserved antigenicity for all cases.

In conclusion, even though the loss of 5hmC immunoreactivity may signify *TERT* promoter mutations in subsets of FTCs, we could not prove its clinical value to predict the *TERT* promoter mutational status in this tumor entity. Further studies are therefore warranted. However, the promising role of 5hmC screening of PTCs with synergistic *TERT* promoter and *BRAF* mutations was validated.
